# Spatial Bridge Locking Fixator *versus* Traditional Locking Plates in Treating AO/OTA 32‐A3.2 Fracture: Finite Element Analysis and Biomechanical Evaluation

**DOI:** 10.1111/os.13308

**Published:** 2022-06-22

**Authors:** Jianwei Hu, Ye Peng, Jiantao Li, Ming Li, Ying Xiong, Jiayu Xiao, Licheng Zhang, Peifu Tang

**Affiliations:** ^1^ Department of Orthopaedics, First Medical Center, Chinese PLA General Hospital National Clinical Research Center for Orthopedics, Sports Medicine & Rehabilitation Beijing China; ^2^ Department of Orthopaedics Tangshan Gongren Hospital Tangshan China; ^3^ Department of Orthopaedics Yan'an Hospital Affiliated to Kunming Medical University Kunming China

**Keywords:** biomechanics, femoral shaft fracture, finite element analysis, interfragmentary movement, secondary healing

## Abstract

**Objective:**

To compare the biomechanical behaviors of the spatial bridge locking fixator (SBLF), single locking plate (SP), and double locking plate (DP) for AO/OTA 32‐A3.2 fractures using finite element analysis and biomechanical tests.

**Methods:**

Axial loading of 700 N was conducted on the AO/OTA 32‐A3.2 model via finite element analysis. The von Mises stress and the interfragmentary movement (IFM) were comparatively analyzed in the three configurations above. On the mechanical tester, axial and torsional loading of 30 synthetic femurs (five specimens of each configuration for each test at random) was performed, and the interfragmentary movement, torsion angle, stiffness, and ultimate load were recorded and analyzed.

**Results:**

The finite element analysis (FEA) results showed that the von Mises stress of the spatial bridge locking fixator (SBLF) was lower than that of the single locking plate (SP) and higher than that of the double locking plate (DP). At 700 N, the axial IFMs were 0.15–0.38 mm (SBLF), 0.03–0.84 mm (SP), and 0.02–0.07 mm (DP). The biomechanical experiment indicated that the axial interfragmentary movements (IFMs) were 0.44 ± 0.23 mm (SBLF), 1.02 ± 0.40 mm (SP), and 0.07 ± 0.07 mm (DP) (*p* < 0.001). The axial IFM of the SBLF group had the highest probability (79.26%) of falling within the ideal range (0.2–0.8 mm), and the SP and DP groups had probabilities of 27.10% and 3.14%, respectively. The axial stiffness in the SBLF group (1586 ± 130 N/mm) was significantly lower than that in the DP group (10,264 ± 2671 N/mm) (*p* < 0.001) but greater than that in the SP group (725 ± 178 N/mm) (*p* = 0.396). The range of axial loads to ultimate failure was 3385–4527 N (SBLF), 3377–4664 N (SP), and 3780–4804 N (DP). The shear motion of the fracture end was 0.35 ± 0.14 mm (SBLF), 0.16 ± 0.10 mm (SP), and 0.08 ± 0.04 mm (DP) (*p* < 0.001). The torsional stiffness was 1.68 ± 0.14 Nm/degree (SBLF), 2.32 ± 0.29 Nm/degree (SP) (SBLF&SP, *p* < 0.001), and 3.53 ± 0.73 Nm/degree (DP) (SBLF&DP, *p* < 0.001).

**Conclusions:**

The SBLF structure may exhibit a better biomechanical performance compared with the SP and DP in providing the best quantity and more symmetrical interfragmentary movement for AO/OTA 32‐A3.2 fractures.

## Graphical abstract

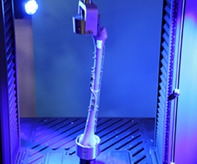

For secondary healing of AO/OTA 32‐A3.2 fracture, the spatial bridge locking fixator system may provide optimal quantity and more symmetrical interfragmentary movement without cortical compression, while effectively reducing stress concentration and stress shielding.

## Introduction

The treatment of long shaft fractures has been one of the classic tasks of orthopaedic surgeons for a long time. A retrospective study from England and Wales showed that the incidence of femoral shaft fractures was 10.3/10^5^/year, accounting for 0.9% of total body fractures^1^, and they are a serious public health problem. The therapies have also evolved based on a better understanding of the local anatomy and biomechanics involved in fixation techniques^2^, ^3^.

There are two main modes of fracture healing: primary (direct) healing and secondary (indirect or spontaneous) healing^4^. Fracture union is actually determined by the mechanical condition of the fracture site. The mechanical factors affecting secondary healing are the stress, interfragmentary movement (IFM), gap (L), and interfragmentary strain (IFS). The quantitative relationship is reflected by the formula “IFS = IFM/L × 100%”^5^. Therefore, it is necessary to generate the most appropriate IFM *via* elastic fixation. The occurrence of more uniform values of IFM between the opposite points at the fracture ends helps ensure the appearance of high‐quality callus to facilitate fracture healing.

Simple fractures are more demanding in terms of the strain at the fracture end. The blood supply and biological environment of the fracture site are the basis for healing. Likewise, the mechanical environment provided by opportune IFM is considered to be the key to promoting callus healing. Moreover, the symmetry of the callus directly affects the quality of fracture healing^6^. Extramedullary osteosynthesis has always played a significant role and will not be replaced in the future^2^. However, as the classic representative of extramedullary fixation, the plates have unavoidable drawbacks. For example, the strategy of treating simple diaphysis fractures with traditional compression plates has long been challenging. Anatomical reduction and rigid fixation by compression plates or lag screws may be responsible for stress shielding, cortical necrosis, and stress concentration due to excessive stiffness^7^, ^8^, which have consistently demonstrated nonunion rates >10%^9^. The concept of biological osteosynthesis (BO) has promoted the development of locking plates and bridging osteogenesis technology^10^. However, they are significantly improved but still flawed; single locking plate fixation has the disadvantages of asymmetric callus, and bridge fixation can sometimes lead to excessive elasticity. The reason is that the bending of the plate is a major cause of axial micromotion at the fracture site in plate bridging fixation, and the micromotion of the fracture site beneath the plate is obviously less than that on the opposite side, resulting in asymmetric callus formation and rendering it difficult to accurately regulate and control the system stiffness, usually resulting in excessive elasticity^11^. Excessive elasticity may lead to instability and nonunion, with a reported nonunion rate of 20%^12^, ^13^. Improved far cortical locking (FCL) techniques and dynamic locking plates (DLPs) are improvements that still cannot provide the most appropriate IFM for callus union^14^. As a result, double plates are often used to enhance stability. In contrast, their rigidity is too large to produce adequate and symmetrical calli, and osteogenesis can only be accomplished by the crawling replacement of bone tissue after a long period of steel fixation^2^.

Intramedullary nails have become the first choice for the treatment of simple fractures of the long shaft, providing intramedullary fixation without damaging the outer periosteum and blood supply^15^, ^16^. They can be implanted in such a way that surgical injury and other complications are minimized. However, intramedullary osteosynthesis is not perfect because of its limitations, such as the oppression of the endosteum, single configuration, mismatch with the femoral anterior arch, damage of the epiphyseal, and limited use in patients with an excessively small medullary cavity or intramedullary prosthesis. Moreover, the amount and direction of the IFM is not easy to control^2^, ^17^, ^18^. In addition, the nonunion rate was 10%^19^, and the complication rate was 20.5%^20^. Most importantly, implants that can accurately provide the optimal elasticity for the healing of simple fractures will become mainstream in the future.

**Fig. 1:**
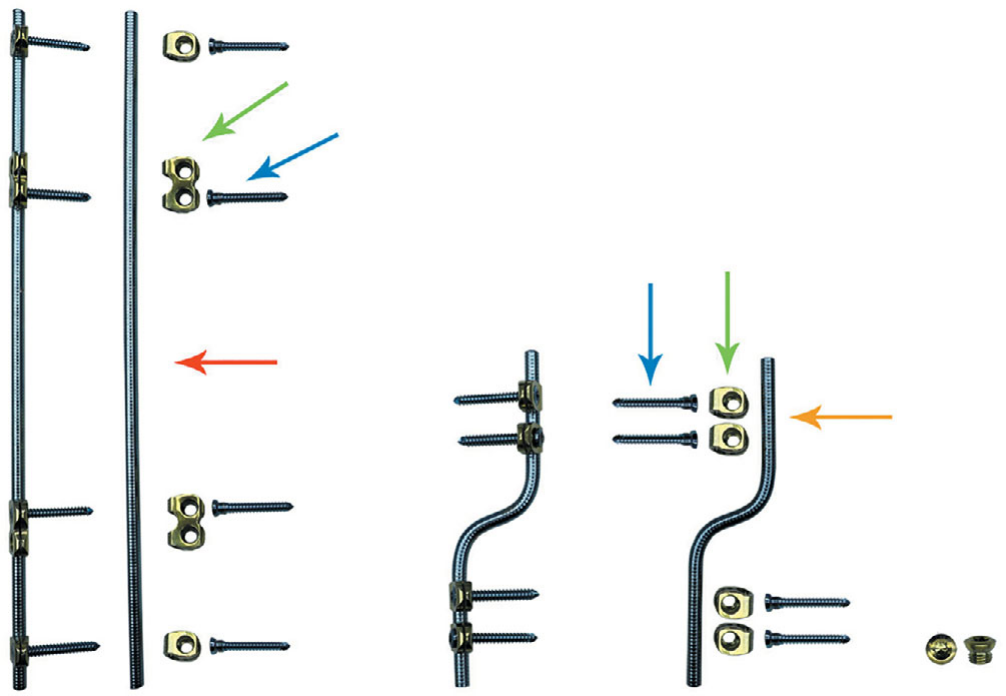
Spatial bridge locking fixator (SBLF) that consists of the bridge rods, the locking screws, and the locking clips. The bars comprise of a long straight rod (red arrow) and a short sigmoid rod (orange arrow), both of which are framed to the surface of the femoral shaft by the locking screws (blue arrow), threading through the clips (green arrow), but not in contact with the shaft

Therefore, we designed a new internal fixation device based on the anatomical and biomechanical characteristics of the femoral shaft. It is expected to avoid the disadvantages of the aforementioned implants and retain the original advantages further, sequentially achieve a spatial fixation that provides the most suitable elasticity, ensure more uniform callus formation, and keep blood supply intact. The device is called the spatial bridge locking fixator (SBLF) (Figure 1). The SBLF system consists of bridge rods, locking screws, and locking clips. The rods comprise of a long straight rod and a short sigmoid rod, both of which are framed to the surface of the femoral shaft by locking screws threading through the clips but not in contact with the shaft.

Obviously, it is necessary to compare the biomechanical behaviors of SBLF with locking plates and intramedullary nails. Therefore, the purpose of this study was (i) as part of the above comparative study, to investigate the biomechanical performance of a SBLF compared with a SP and DP by finite element analysis and biomechanical testing and (ii) to confirm that the new configuration perfectly fits the anatomical and mechanical characteristics of the femoral shaft, enabling the fracture end to produce the most appropriate and symmetrical displacement in the AO/OTA 32‐A3.2 fracture type. We hypothesized that the SBLF has a greater biomechanical advantage than the locking plates in the fracture type corresponding to AO/OTA 32‐A3.2 fractures.

## Materials and Methods

### Finite Element Analysis Study

**Fig. 2:**
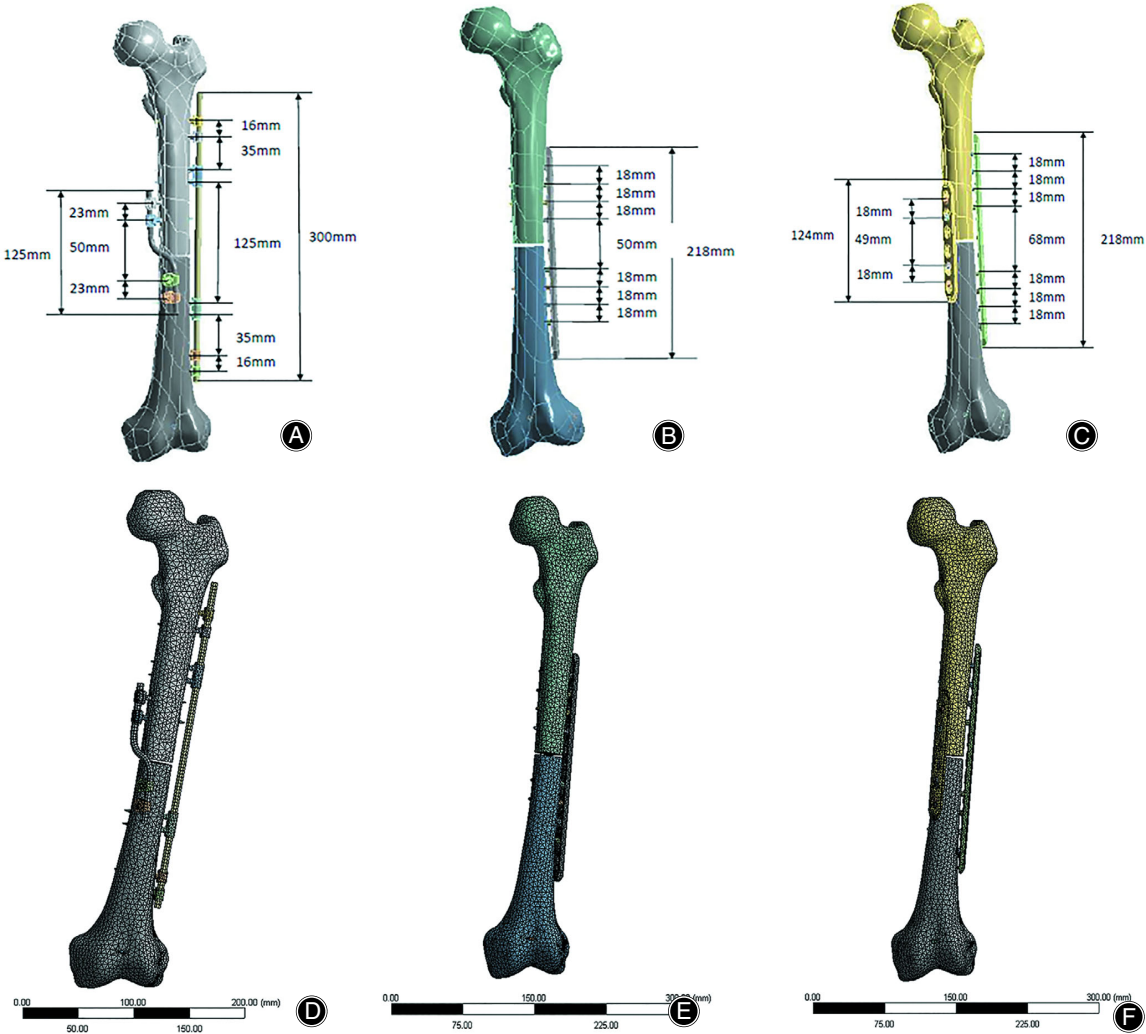
The 3D model of AO/OTA 32‐A3.2 fracture with implants and their geometric parameters: (A) the spatial bridge locking fixator (SBLF) system, (B) the single locking plate (SP) system, (C) the double locking plate (DP) system. Finite element model of AO/OTA 32‐A3.2 fracture with implants was created in the software of Workbench 15.0: (D) SBLF system, (E) SP system, (F) the DP system

The geometric model was derived from three‐dimensional CT scan reconstruction data of a fourth‐generation composite femur (Model 3403, Sawbones, Vashon Island, WA). Subsequently, the fracture model in PTC CREO 2.0 (PTC Inc., USA) was constructed according to Giordano *et al*.^2^, ^17^, and a transverse 3‐mm osteotomy was performed at the midpoint of the femoral shaft perpendicular to the anatomical axis to simulate the AO/OTA 32‐A3.2 fracture (Figure 2).

According to the engineering drawing provided by the manufacturer, PTC CREO 2.0 software (PTC Inc.) was used to generate the 3D models of the three internal fixation configurations. The SBLF system consists of a bridge rod, locking screw, and locking clip. The bars are framed to not be in contact with the shaft. The SBLF (Bridge system, fixed block 02: QZX04‐02‐01; connecting rod 05: QZX01‐05, diameter 6.0 mm; locking screw: QZX02‐02‐01; materials: TC4; Tianjin Walkman Biomaterials Co., Ltd., China) geometric parameters, screw distribution and position are shown in Figure 2A. The working length of the short sigmoid rod was 50 mm (the span between two nails closest to the fracture site on the short sigmoid rod), and its working width was 30 mm (the distance between the straight parts of the sigmoid rod). The distal end of the anteromedial sigmoid rod and the lateral straight rod were distributed at an angle of 90° along the circumference of the cross section. The straight rod was 300 mm long with a 125‐mm working length, and the two ends could be deformed in line with the shape of the femoral epiphysis. According to previous research results^21^, ^22^ and clinical experience^23^, the 11‐hole locking compression plate (LCP, Tianjin Walkman Biomaterials Co., Ltd. China) (width = 17.5 mm, thickness = 5.5 mm) was used to complete the bridging fixation of a single plate (Figure 2B). The DP fixation system consists of a lateral 11‐hole plate (ditto) and an anteromedial short six‐hole plate (LCP, Tianjin Walkman Biomaterials Co., Ltd.) (width = 16 mm, thickness = 3.5 mm) (Figure 2C). Assembly of the implants and bones was performed in PTC CREO 2.0 (Figure 2A–C).

The assembled 3D models were imported into Workbench 15.0 (ANSYS. CORP, USA) to generate the finite element model (Figure 2D–F). The femur model was assumed to be homogeneous and isotropic with linear elasticity, and the mechanical properties of the implant and bone materials are shown in Table 1. This was determined by the manufacturer and previous studies^24^.

**TABLE 1:** Mechanical features of the implant and bone

Material	Elastic modulus (GPa)	Poisson's ratio	Mesh size (mm)
Cortical bone	17.0	0.30	2.5
Cancellous bone	0.7	0.29	2.0
Locking clip	110	0.33	1.5
Bridging rod	110	0.33	1.5
Locking screw	110	0.33	1.5
LP	110	0.33	1.5

Abbreviation: LP, locking plates.

The interface between bone and implant was simulated by a contact pair with a friction coefficient of 0.3, and the cortical‐cancellous bone was set as bonded. The interface between the bar and the locking clip of SBLF was set as tie constraints. All nodes on the distal surface of the femur were constrained to 0 degrees of freedom. The proximal femur was free to rotate around the anteroposterior central axis of the femoral head under the load^25^. The study simulated the forces on one person's limb while upright, which was applied to the femoral head at 9° posteriorly on the sagittal plane and 11° laterally in the coronal plane. In this study, the femoral head was loaded to 700 N to imitate the physiological state of people standing on one leg.

**Fig. 3:**
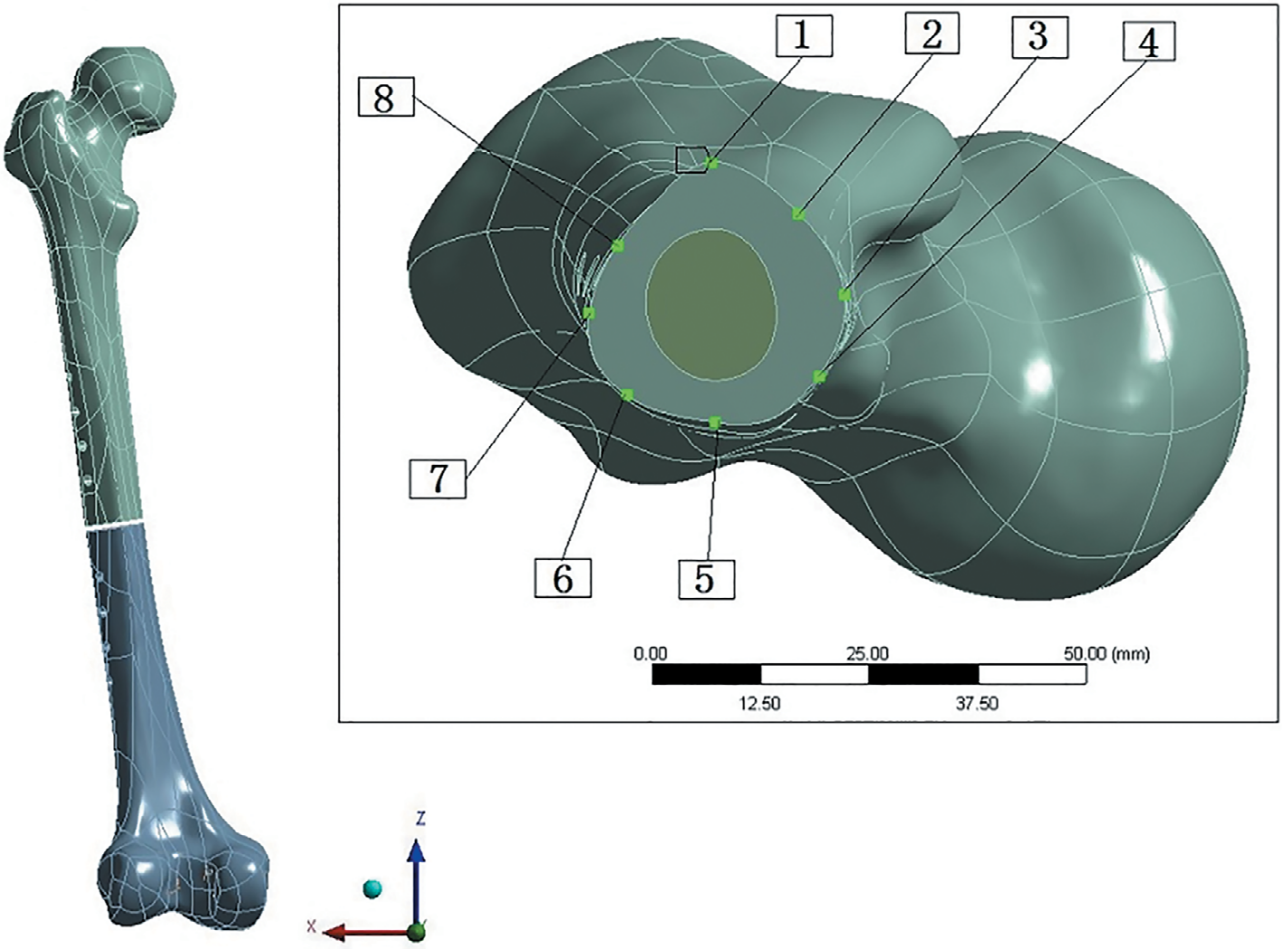
The simulation diagram of section shows the markers

Finite element software Workbench 15.0 was used for analysis. The von Mises stress distribution and the Interfragmentary Movement (IFM) on the three quadrants were used to compare the effect of the fixation structure. Eight points uniformly distributed on the fracture section edge of the proximal fragment were used to capture the mechanical factors (Figure 3).

### Biomechanical Tests

#### Preparation and Osteotomy

Thirty fourth‐generation composite femurs (Model 3403, Sawbones, Vashon Island, WA) were randomly divided into three groups, namely the SBLF, SP, and DP groups (15 in axial loading tests and 15 in torsional loading tests). Prior to osteotomy, the implant was fixed to the synthetic femur. To minimize the differences between samples, the fixation procedure was consistent, and a torque of 10 Nm was applied to each screw^26^.

**Fig. 4:**
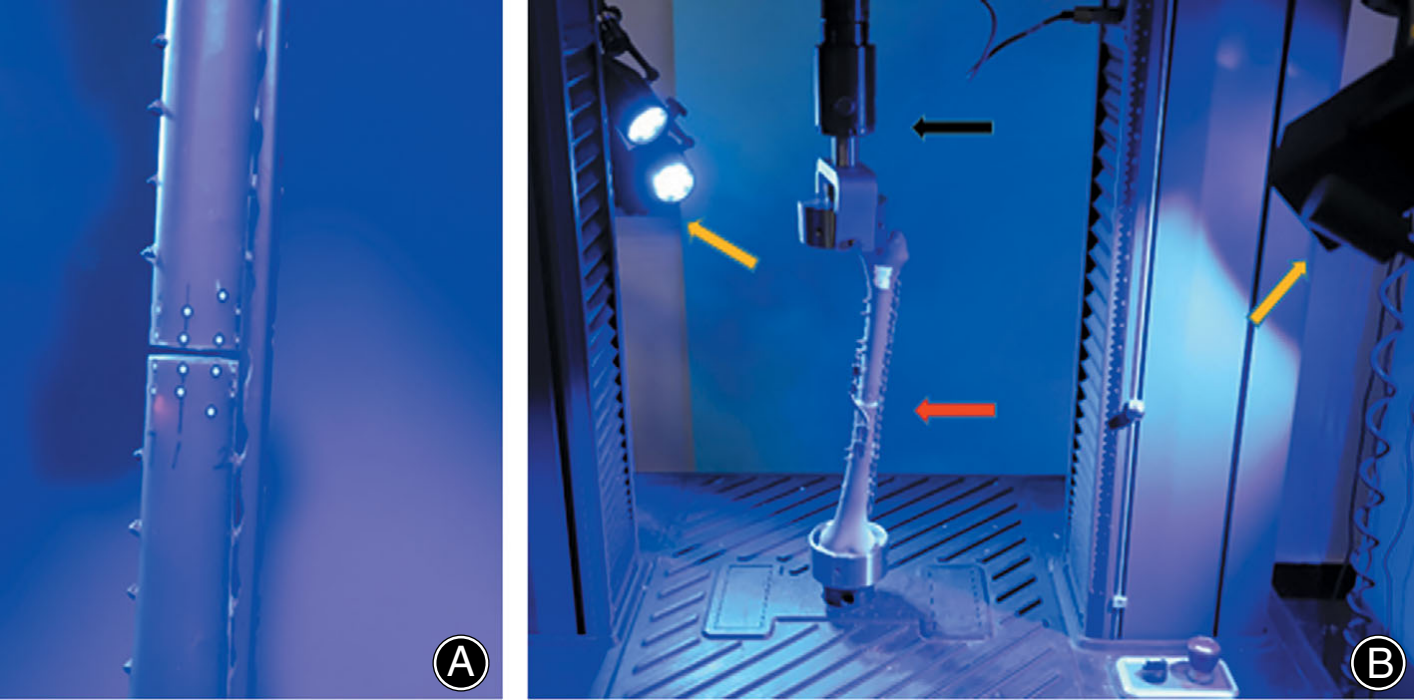
(A) Local observation of osteotomy section with markers. (B) The completed sample with implants (red arrow) was installed on the mechanical testing machine (black arrow). Two cameras (orange arrow) of optical measuring systems could be seen in front and in the back

A swing saw (Bojin Machine Tool Co., Ltd., Shanghai, China) was used to obtain the fracture line perpendicular to the anatomical axis of the femur at 21 and 21.3 cm from the apex of the greater trochanter^2^, ^27^. Six pairs of representative and homodispersed points along the edge of the fracture line were affixed markers (Figure 4A), and the IFM of all marked points was captured using the optical measurement system Aramis 3D 12 M (GOM, Braunschweig, Germany) (Figure 4B). All samples were prepared by the same investigator according to the manufacturer's surgical technique.

#### Fixation

Implant fixation was performed again after osteotomy. The three configurations in the mechanical experiments were completely consistent with the finite element model. The femoral head and 4.5‐cm‐long distal femur were embedded into a custom Denture Base Resin (Dajin Dental Materials Co., Ltd., Kunshan City, China) steel cylinder. A custom alignment device was used during the embedding of the distal and proximal femur to ensure that the axial compression loading direction was consistent with the mechanical axis of the femur and that the implants would not come into contact with the fixtures during loading. The axial load is transmitted through a custom‐hinged device with an axostylus passing through the sagittal central axis of the femoral head. The specimens were adducted 11° in the coronal plane and vertical in the sagittal plane to simulate a single leg stance^27^, ^28^.

#### Axial Loading Tests

Axial static loading tests (15 specimens, five for each configuration) were conducted on an MTS machine (Exceed TM Model E45, MTS (China) Co., Ltd; Figure 4B). In the present study, the load was performed as described by previous researchers^28^. The vertical load was applied to the femur at a rate of 2 mm/s with an initial load of 50 N. Failure was defined as a failure of the implant, a fracture of the femur, or a 30% drop in load corresponding to an irreversible negative slope observed on the load–displacement curve^29^. The load when failure occurs was defined as the yield load. The mean linear slope of the elastic part of the curve was defined as the stiffness. If the fracture ends contact, the loading continues until the ultimate failure occurs.

In the axial compression load test, in addition to the comparison of stiffness, a multiperspective analysis of the IFM was also conducted under the 700 N load, which corresponds to the load on one femur when a person stands. These indices included the coefficient of variation (CV) of the IFM, the probability of the axial IFM falling into the ideal range (0.2–0.8 mm), the shear micromotions of the section, and the ultimate load.

#### Torsion Loading Tests

To assess the torsional load (15 specimens, five for each configuration), the composite femurs were potted at both ends in cylindrical denture base resin cups and then fixed horizontally on the torsional test machine (ND‐200, Changchun Kexin Test Instrument Co., Ltd. Changchun, China), which would allow the axis of torque to be unified with the anatomical axis of the femoral shaft. Rotational forces were applied proximally. Each construct was loaded to 10 Nm at a rate of 60°/min. Load displacement curves were generated, and stiffness was calculated as the slope of the linear portion of the curve.

#### Optical Measuring System

**Fig. 5:**
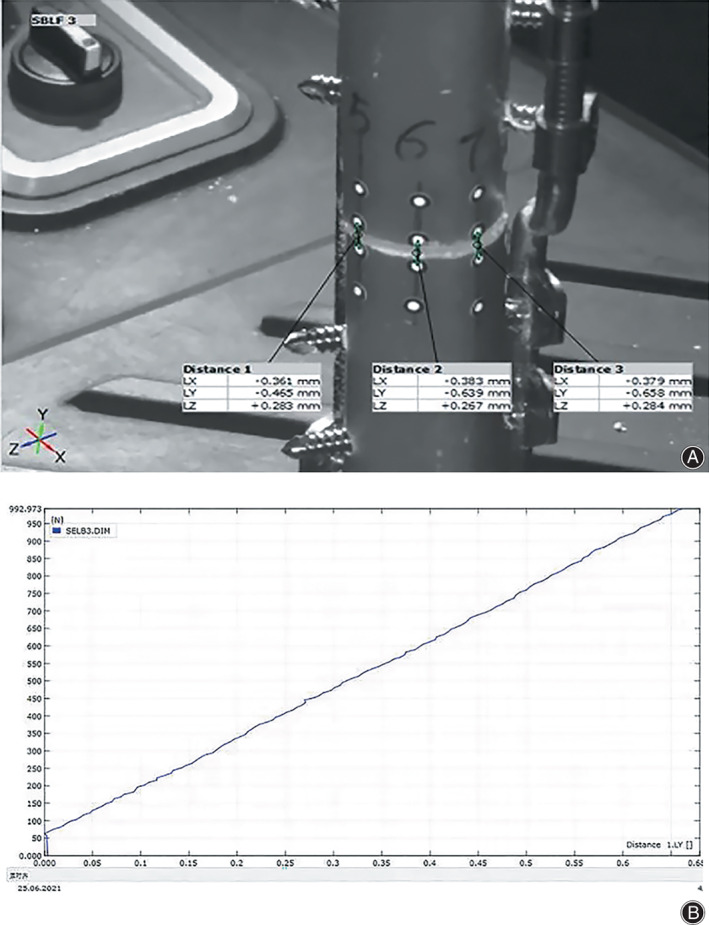
The result graph automatically generated with ARAMIS Professional: (A) video snapshot, (B) load displacement curve in compression direction

Two sets of optical measuring systems, ARAMIS 3D 12 M (GOM, Braunschweig, Germany), including two specialized digital cameras, were used to view the focus on the end of the fracture from different angles to assess the maximum range (Figure 4B). The optical system was parallel to the MTS through the same time node. The optical system captured the relative micromotion of the markers at each load level at a frequency of 2 Hz. Aramis software was used to process the acquired images and original data, and the results were presented in the form of deformation animation, multiview photographs and load–displacement curves (Figure 5).

### Statistical Analysis

SPSS (version 18.0, SPSS Inc., Chicago, IL) was used for data analysis. All tests were carried out under uniform conditions. The data were first tested to assess normality (Shapiro–Wilk Test) and the homogeneity of variances between each group (Levene Test). The measurement data were compared by ANOVA. Mechanical parameters of each group were expressed as the mean ± standard deviation (mean ± SD), and the significance level was determined as *p* < 0.05. *p* < 0.001 was used when the *p* value was <0.001. Based on the results of the pre‐experimental data and data from previous studies, the minimum sample size required for each index was estimated (*α* = 0.05, 1 − *β* = 0.90). Among them, a one‐tailed test was adopted for IFM and axial stiffness, while a two‐tailed test was used for shear micromotions and torsional stiffness. According to the estimated results, 30 elements per group (five samples with six observation sites in each case) were used in this experiment to ensure its statistical efficacy.

## Results

### FEA Results

The number of nodes and elements in the model is shown in Table 1.

### The von Mises Stress Distribution

**Fig. 6:**
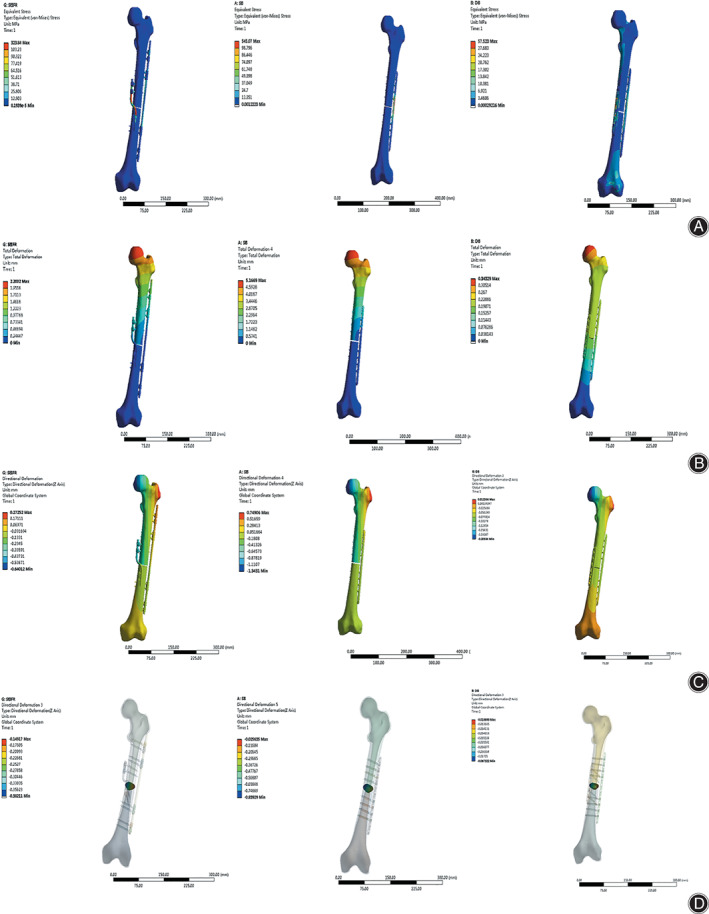
Contour plots of von Mises stress of the three plants systems (A), the model total deformation (three‐DOF) of the three plants systems (B), the compression deformation (vertical axis) of the three plants systems (C), the axial interfragmentary movement (IFM) of the three plants (D), with respect to 700 N loads in three implant groups. Note: DOF, degrees of freedom

The stress of the SBLF structure was mainly concentrated on the bend of the sigmoid bar, with a maximum value of ~323.84 MPa (Figure 6A). The stress of the SP structure was concentrated on the bridge segment of the plate across the fracture site, and the maximum stress was ~545.07 MPa. For the DP structure, the maximum value appeared on the short plate, which was ~57.52 MPa. FEA showed that the maximum stress of the SBLF structure was between that of the SP and DP structures.

### Interfragmentary Movement (IFM)

With increasing load, the relative displacements of the three implant systems are shown in Fig. 6B–D. The maximum displacement occurred on the femoral head. In terms of the axial IFM at 700 N, the range of the SBLF system (0.15–0.38 mm) was between that of the SP system (0.03–0.84 mm) and the DP system (0.02–0.07 mm).

With regard to the shear displacements of the fracture site, combined analysis of the X and Y axes was performed. The data are as follows: 0.03–0.21 mm (SBLF), 0.12–0.27 mm (SP), and 0.10–0.17 mm (DP).

### Biomechanical Results

#### Axial Test

**Fig. 7:**
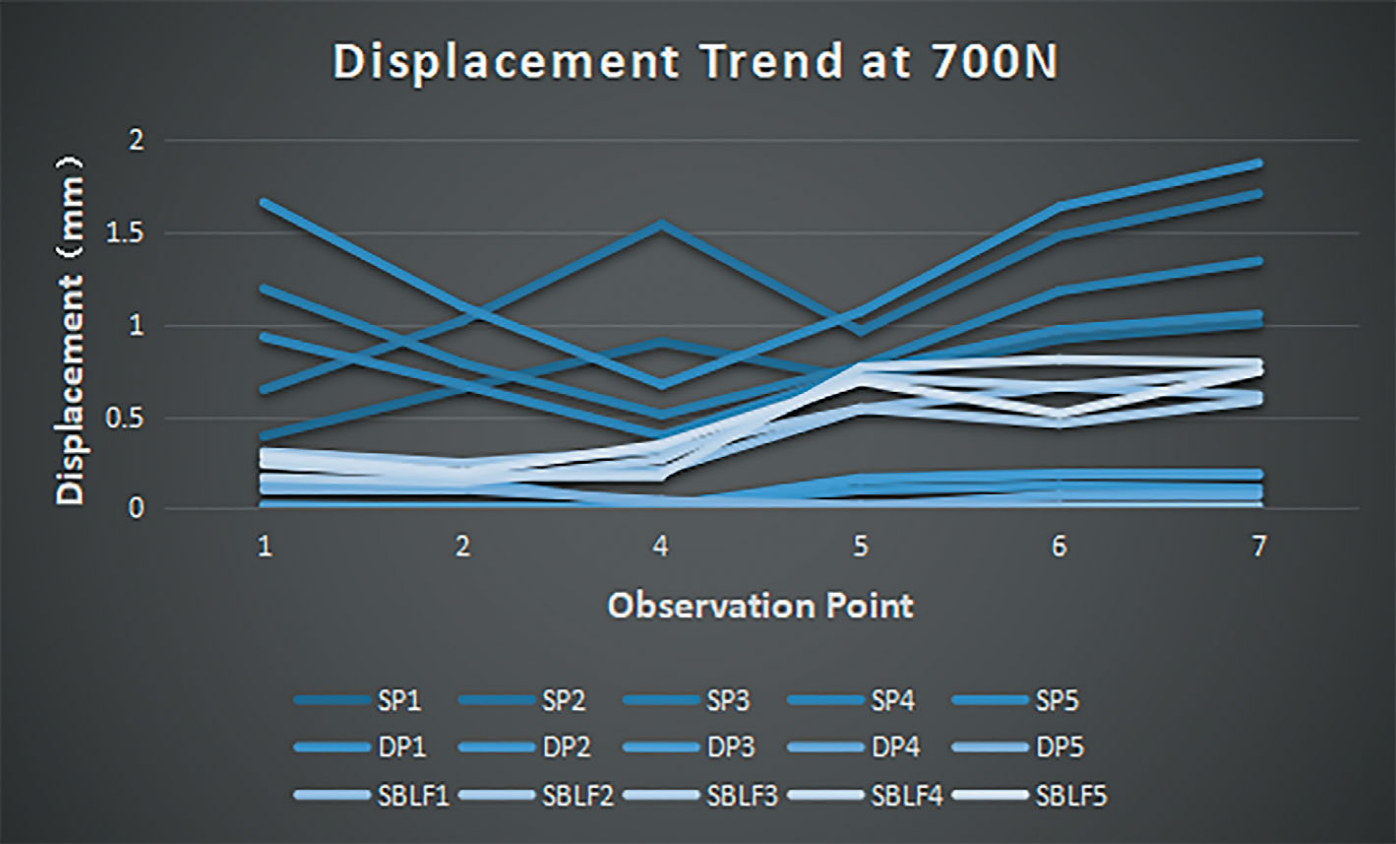
Axial IFM trend of three groups of constructs at 700 N. Each group of colors corresponds to the displacement ranges of each configuration occupying significantly different partitions. It was clearly displayed that the SBLF group mostly fell in the ideal range (0.2–0.8 mm)

Similarly, at a 700‐N load, the axial IFM in the SBLF group (0.44 ± 0.23 mm) was intermediate compared with the SP group (1.02 ± 0.40 mm) (*p* < 0.001) and the DP group (0.07 ± 0.07 mm) (*p* < 0.001) (Table 2). With respect to the CVs of the axial mean IFM, the value in the SBLF group (0.52) was greater than that in the SP group (0.39) and significantly smaller than that in the DP group (1.0). The probability of axial IFM of the SBLF construct falling into the ideal range (0.2–0.8 mm) at a 700‐N load was 79.26% compared with the SP construct (27.10%) and DP construct (3.14%). The displacement distribution trend of each observation point of the three constructs at 700 N is shown in Fig. 7. The stiffness in the SBLF group (1586 ± 130 N/mm) was significantly lower than that in the DP group (10,264 ± 2671 N/mm) (*p* < 0.001) but greater than that in the SP group (725 ± 178 N/mm), but the difference was not statistically significant (*p* = 0.396).

**TABLE 2:** Data analysis of axial IFM (mean ± SD)

	SBLF	SP	DP
Axial IFM (mm)	0.44 ± 0.23	1.02 ± 0.40	0.07 ± 0.07
Significant Difference	–	*p* < 0.001 (SP&SBLF)	*p* < 0.001 (DP&SBLF)
Coefficient of Variation	0.52	0.39	1.0
95% CI of the Difference (mm)	0.36–0.53	0.87–1.16	0.05–0.10
Probability in 0.2–0.8 mm (Significant Difference)	79.26%	27.10% (*p* < 0.001, SP&SBLF)	3.14% (*p* < 0.001, DP&SBLF)

*Notes*: ANOVA of the three groups showed statistically significant differences, *p* < 0.001(SBLF&SP or SBLF&DP). The probability of falling within the ideal range (0.2–0.8 mm) is 79.26% (SBLF), 27.10% (SP) and 3.14% (DP), respectively (*p* < 0.001).

Abbreviations: IFM, interfragmentary movement; CI, confidence interval, SBLF, spatial bridge locking fixator; SP, single locking plate; DP, double locking plates.

Correspondingly, the shear micromotions of the three groups were 0.35 ± 0.14 mm (SBLF), 0.16 ± 0.10 mm (SP), and 0.08 ± 0.04 mm (DP). The differences were all statistically significant (*p* < 0.001), and all the shear micromotions were acceptable.

#### Static Failure Test

**Fig. 8:**
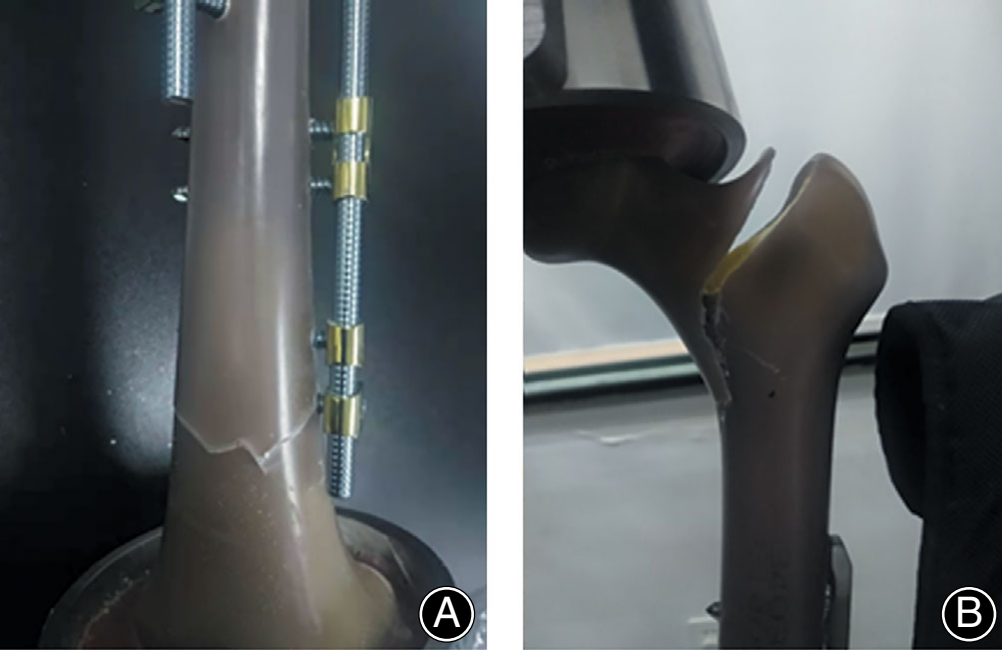
Compression load failure: (A) the faulted farthest screw hole with SBLF fixation, (B) femoral neck fracture with DP fixation

In the SBLF group, osseous contact occurred under a load of 2600–3500 N, and that in the SP and DP groups corresponded to 2900–3700 N and 3000–4300 N, respectively. Upon loading after contact, the ranges of axial loads to ultimate failure of the three configurations were 3385–4527 N (SBLF), 3377–4664 N (SP), and 3780–4804 N (DP) (Figure 8). They were all above the safety threshold for postoperative loads.

#### Torsion Test

The torsional stiffnesses of the three constructs were 1.68 ± 0.14 Nm/degree (SBLF), 2.32 ± 0.29 Nm/degree (SP) (SBLF&SP, *p* < 0.001), and 3.53 ± 0.73 Nm/degree (DP) (SBLF&DP, *p* < 0.001).

## Discussion

### Superior Performance over Traditional Locking Plates

The present study established a new concept of internal fixation, based on which a new implant for AO/OTA 32‐A3.2 fractures was introduced, and its advantages were demonstrated. Although it is an extramedullary fixator, it is different from traditional eccentric fixation, and its excellent mechanical properties can provide the most suitable and symmetrical axial IFM for callus healing. The FEA and biomechanical test both showed that the SBLF fixation system performed better than the SP and DP systems in terms of IFM symmetry. Most importantly, it was precisely located in the optimal area for callus healing (0.2–0.8 mm) at a 700‐N axial load, which was verified by previous experiments and clinical experience^28^, ^30^, ^31^, ^32^. In the static strength test, there was no implant destruction, but fractures of the synthetic bone were found (Figure 8). Tsai *et al*. obtained a similar static failure mode in plate structures^26^. The results of FEA and biomechanical testing were consistent in that the elasticity of the SBLF system was between that of the SP and DP systems. Our aim was to obtain the trend rather than the absolute value by FEA. The trends of the two methods were consistent, which reinforces the reliability of the results. To the best of our knowledge, this is the first study to investigate the biomechanical advantages of the SBLF construct over the SP and DP constructs in AO/OTA 32‐A3.2 fractures by FEA and biomechanical tests.

Coefficient of variation (CV) was of reference significance to the symmetry of relative displacement on the fracture site. However, for this study, regardless of how small the CV was, if the mean did not fall within the optimal range, it was not conducive to the formation of superior callus. Therefore, this study introduced a more critical index, which was the probability of axial motion falling into the ideal range. Numerous experiments and clinical practices have proven that the area with a core range of 0.2–0.8 mm is both safe and effective^28^, ^30^, ^31^, ^32^.

### SBLF Perfectly Fits the Femoral Shaft Characteristics

The AO/OTA 32‐A3.2 fracture was characterized by simple transverse fractures of the femoral shaft with less strain tolerance than comminuted fractures. Because the comminuted fracture had many bone fragments dispersing strain, the strain was concentrated at one fracture site of the simple fracture. If the strain was too small, the callus could not be activated and could only complete the primary healing. If the strain was too large, the callus could be generated, but it was difficult to ossify and led to nonunion. As a result, for simple fractures, the control of the strain at the fracture site should be very precise.

The geometry of the femur was known to be irregular, and its anatomical and mechanical axes were not uniform. The proximal end of the sigmoid bar was away from the lateral straight bar, and the distal end was near the lateral bar. This enabled the femur with irregular geometry partially to offset the original lateral tension and medial compression under the combined effect of the lateral straight rod and the anteromedial sigmoid rod when the external force was directed along the femoral mechanical axis such that the IFM of all parts of the fracture section tended to be symmetrical.

### Flexible Combination to Adjustable Elasticity

This study only extracted the data under a 700‐N compression load for analysis because 700 N represents the average human weight of 70 kg. This imitated the physiological state of people standing on one leg, which has often been used as a load condition in biomechanical experiments^26^, ^29^, ^30^, ^33^, ^34^. Moreover, it is significant to note that the stiffness of the SBLF system was adjustable because of the characteristics of its constituent elements, which facilitated the preoperative selection of parameters for individual weight and postoperative activity, such as rod diameter, working width, and working length. In addition, it could be inserted percutaneously, which protects the blood supply of the fracture site. In addition, it was not like a plate pressing on the bone cortex but more similar to an external fixator implanted in the body.

### Research Method Innovation

The present study adopted the following innovative methods: (1) Local parameters of the fracture site were selected as the research object, which affect fracture healing. Most previous studies have measured the strain or deformation of the entire femur using a contact strain gauge or sensor^27^, which does not accurately reflect the situation at the breakpoint. In addition, the displacement at some specific points could not be measured with the previous methods^10^, ^26^. The contactless optical measurement and FEA used in the present study are the two most advanced methods^28^. The core aspect of FEA is spatial discretization. This structural analysis enables the determination of stresses caused by external forces, pressures, and other factors. The von Mises strain diagram is often used in the field of FEA to evaluate the stress distribution, which is equivalent to the equivalent stress, also known as stress intensity. Finite elements can measure areas that cannot be assessed by experiments, such as the bone cortex beneath the implant. Since the FEA simplified the material properties, structure, and load of bone, the advantages of the SBLF system obtained by FEA were also verified by mechanical experiments. The FEA results of the plate were similar to those of previous studies^21^. Furthermore, the biomechanical values in this study were similar to those recorded in previous studies involving plates^26^, ^35^. It should also be stated that due to the barrier of the plate itself, the IFM below the plate was difficult to measure in the mechanical experiment. Consequently, the actual minimum displacement of the fracture site in the plate group was larger than that in the real situation, which undoubtedly artificially exaggerates the symmetry. Specifically, in terms of symmetry, the advantage of the SBLF system was actually greater than the measured value. (2) When measuring the IFM and IFS in the mechanical experiment, an advanced optical measuring system was used to make the positioning of the measured part more precise, the data more accurate, and the manipulation more convenient.

### Limitations

Nevertheless, the present study has several limitations. First, this study is the first part of a comparative study, so there was no comparison available with the internal fixation configuration, such as intramedullary nailing, which has been the gold standard for the treatment of simple fractures of the long shaft. Second, bending tests and cyclic loading tests were not carried out in this experiment. Third, this experiment collated the data at 700 N for comparison to imitate the physiological state of people standing on one leg, which represents the average human weight of 70 kg. The parameters under different loads should be analyzed systematically and in more detail. Fourth, no controlled comparison of animal and clinical trials was conducted in this study. Researchers need a more continuous analysis and comparison to have a comprehensive and objective understanding of SBLF performance and characteristics.

## Conclusion

In conclusion, compared with SPs and DPs, the SBLF system may provide the best quality of IFM and more symmetrical IFM without cortical compression while effectively reducing stress concentration and stress shielding. The SBLF system is effective and feasible for secondary healing of AO/OTA 32‐A3.2 fractures.

## AUTHOR CONTRIBUTIONS

Jianwei Hu: designed the study; performed the experiments; collected and analyzed the data; drafted and revised the manuscript. Ye Peng: performed the experiments; collected, analyzed, and interpreted the data; revised the manuscript. Jiantao Li: collected, analyzed, and interpreted the data; read and approved the final manuscript. Ming Li: collected the data; read and approved the final manuscript. Ying Xiong: designed implant configuration; read and approved the final manuscript. Jiayu Xiao: designed implant configuration; read and approved the final manuscript. Licheng Zhang: designed the study; revised the manuscript. Peifu Tang: supervised the study.
